# Low Dose Isoflurane Exerts Opposing Effects on Neuronal Network Excitability in Neocortex and Hippocampus

**DOI:** 10.1371/journal.pone.0039346

**Published:** 2012-06-18

**Authors:** Klaus Becker, Matthias Eder, Andreas Ranft, Ludwig von Meyer, Walter Zieglgänsberger, Eberhard Kochs, Hans-Ulrich Dodt

**Affiliations:** 1 Department of Bioelectronics, FKE, Vienna University of Technology, Vienna, Austria; 2 Center for Brain Research, Medical University of Vienna, Vienna, Austria; 3 Max Planck Institute of Psychiatry, Munich, Germany; 4 Department of Anaesthesiology, Klinikum rechts der Isar, Munich, Germany; 5 Institute for Forensic Medicine, Ludwig-Maximilian University of Munich, Munich, Germany; Univ. Kentucky, United States of America

## Abstract

The anesthetic excitement phase occurring during induction of anesthesia with volatile anesthetics is a well-known phenomenon in clinical practice. However, the physiological mechanisms underlying anesthetic-induced excitation are still unclear. Here we provide evidence from *in vitro* experiments performed on rat brain slices that the general anesthetic isoflurane at a concentration of about 0.1 mM can enhance neuronal network excitability in the hippocampus, while simultaneously reducing it in the neocortex. In contrast, isoflurane tissue concentrations above 0.3 mM expectedly caused a pronounced reduction in both brain regions. Neuronal network excitability was assessed by combining simultaneous multisite stimulation via a multielectrode array with recording intrinsic optical signals as a measure of neuronal population activity.

## Introduction

Anesthetics can cause transient arousal and excitation especially during inhalation induction of anesthesia. The excitement phase has first been described as stage II anesthesia for diethyl ether [1] but also occurs with modern volatile anesthetics, including isoflurane [2]–[5]. This phenomenon is in contradiction with the notion that neuronal excitability is dampened during general anesthesia. In fact, one should expect an increased excitability in distinct parts of the central nervous system (CNS). This assumption is supported by the observation that low isoflurane concentrations can accentuate electroencephalographic signs of alertness [6].

Motor excitation of rats during induction of anesthesia with isoflurane is minimized after inactivation of the hippocampal CA1 region [7]. This indicates that, beside the neocortex, also the hippocampus is involved in anesthetic-induced excitation. However, the contribution of the individual subregions to this phenomenon is still largely unknown. To address this question, *in vitro* studies seem to be well-suited [8].

In the present study, we performed measurements of the intrinsic optical signal (IOS) as an indicator of neuronal network excitability (NNE). In brain slices, electrical stimulation induces minimal changes in light scattering and optical transparency. These changes are well-correlated with the spatial extent of neuronal excitation and depend on action potentials [9]–[11]. The tiny variations in optical properties of spiking neurons can best be detected by infrared microscopy and visualized by computer-based image processing [9]. For an optimal comparison of different brain regions with regard to their susceptibility to isoflurane, they should be monitored at the same time in a single brain slice. In order to accomplish this, we used a multielectrode array (MEA) for selective and simultaneous stimulation of multiple anatomical targets. Since the hippocampus seems important in mediating the arousal occurring during stage II anesthesia [7], we chose the CA1 output subfield as one stimulation site. Due to the fact that neocortical layer 5 pyramidal neurons significantly contribute to the EEG [12], we further stimulated neocortical layer 5.

## Materials and Methods

### Slice Preparation

Parasagittal brain slices (300 µm thick) were prepared from 22 male Sprague-Dawley rats (postnatal days 14–21) as described by Eder et al. [13]. All steps of the preparation were carried out in ice-cold artificial cerebrospinal fluid (ACSF) oxygenated with carbogen (95% O_2_/5% CO_2_). The ACSF consisted of (in mM): NaCl, 125; KCl, 2.5; NaH_2_PO_4_, 1.25; CaCl_2_, 2; MgCl_2_, 1; NaHCO_3_, 25; glucose, 25; pH 7.4. The slices were incubated in glass vials containing oxygenated ACSF for 60 min at 34°C and subsequently transferred to the recording chamber. The temperature of the recording chamber was maintained at 34°C by a heating-system (Multi-Channel Systems, Reutlingen, Germany) during all experiments. Animal care and euthanasia was done in accordance with the local animal care comities and with the German animal protection law. According to the German animal experiments act, special approval by an ethics committee or an approval number was not required, since all experiments were performed on isolated organs and not on living animals.

### Microscopy

**Figure 1:**
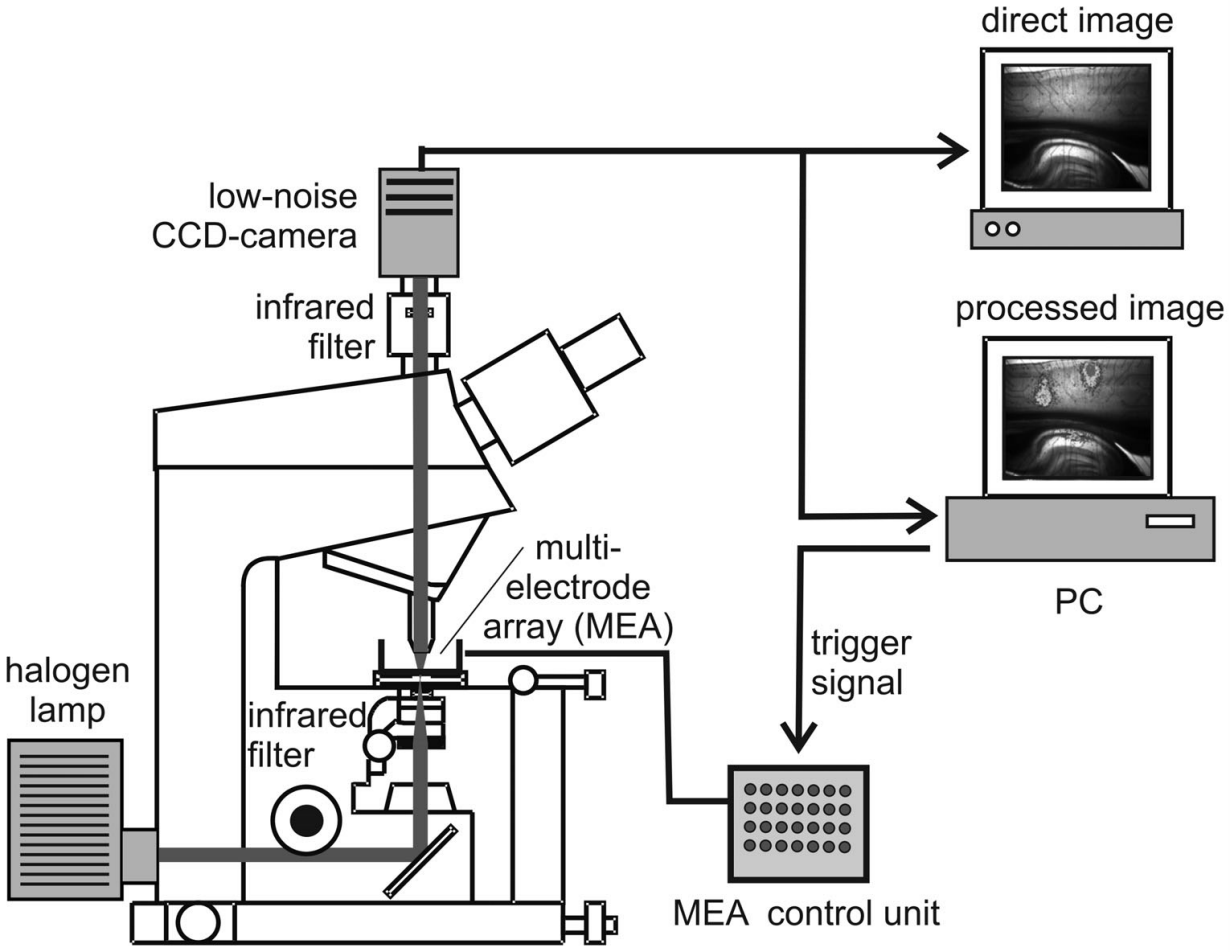
Schematic diagram of the setup used for IOS recordings. The brain slice is superfused with isoflurane-enriched ACSF and electrically stimulated by a MEA. The intensity of the electrical stimuli is separately adjustable for each electrode by a control unit, equipped with 60 potentiometers. The IOS is recorded via an infrared sensitive CCD camera, computationally processed, and subsequently overlaid with a black and white image of the brain slice, thus allowing a correlation with anatomical structures.

Slices were visualized by an upright microscope (Axioskop 2 FS, Zeiss, Göttingen, Germany) with a 2.5x Neofluar objective (N.A. 0.075). To avoid disturbing reflections at the water-air interface, a dipping cone with a transparent cover slip was mounted in front of the objective. All images were obtained in the near infrared spectrum by filtering the light of a 100 W halogen lamp with an optical band-pass filter (λ_max_ = 780±50 nm). To block erroneous stray-light emitted from the room illumination, a second light filter with an identical cutoff frequency was put into the light pathway behind the objective (**Figure 1**).

### Electrical Stimulation

For electrical stimulation, a rectangular 10×6 MEA (Multi-Channel Systems, Reutlingen, Germany) was used (200 µm electrode spacing, 30 µm electrode diameters). The stimulation pulses were generated by a Digitimer 2533 isolated stimulus generator (Digitimer Limited, Herts, UK), which was triggered by a programmable impulse generator board (DAQ PCI 6601, National Instruments, Munich, Germany). The stimulus intensities were varied between 3 and 10 V, and could separately be adjusted for each electrode of the MEA by a control unit equipped with 60 potentiometers. For each electrode of the MEA, the stimulus intensity was adjusted in a way that IOSs of comparable spatial extent and intensity were obtained. In all experiments, the stimulus frequency was 50 Hz and the pulse width was 200 µs. The length of the applied pulse trains was always 2 s. Electrical stimuli were simultaneously applied to neocortical layer 5 and area CA1 of the hippocampus by activating corresponding electrodes of the MEA. Due to a sufficient transparency of the brain slices, individual electrodes of the MEA could easily be localized by visual inspection.

### IOS Recording

The imaging system (**Figure 1**) consisted of a scientific-grade 12-bit CCD-camera (CoolSnap cf, Roper Scientific, Tucson, USA) connected to a computer. Before starting a recording sequence, 10 background images were taken and averaged. Afterwards, the electrical stimulation was started and 10 further images were recorded in intervals of 2 s. The first image was always taken at the onset of stimulation. From each image, the averaged pre-stimulus image was subtracted. To reduce noise and to enhance the visibility of the IOS, the images were low pass filtered by a 5×5 Gaussian filter and subsequently converted into 8-bit pseudo color images. Subsequently, they were computationally overlaid on a black and white image of the brain slice taken before the onset of stimulation.

### Data Analysis

For data analysis, the pixel intensities of the IOS images (12-bit resolution) obtained 4 s after onset of stimulation were integrated within a rectangular bounding box around the respective stimulation electrode, leading to a numerical quantifier for each evoked IOS. All IOS intensities were normalized to the mean IOS intensity obtained during minutes 0 to 20 before onset of drug application. To improve the signal-to-noise ratio, intensity values below a 5% threshold were discarded.

### Application of Isoflurane

Isoflurane (Forene, Abott, Wiesbaden, Germany) was dissolved in the ACSF as follows: carbogen gas was passed through a Forene-calibrated vapor (Vapor 19.3, Drägerwerk AG, Lübeck, Germany) at a constant flow rate of 500 ml/min and afterwards bubbled through the ACSF reservoir. Three different dial settings were chosen to achieve three different concentration levels. Before delivering the isoflurane-containing ACSF to the recording chamber via Teflon tubing, we allowed an equilibration time of half an hour.

### Determination of Isoflurane Concentrations

Isoflurane bath concentrations were determined with a Perkin Elmer 8420 headspace gas chromatograph. The separation was performed on a Megabore capillary column (Rtx-1701; 60 m, 0.53 mm ID, 3.0 µm) with helium as carrier gas at 5 ml/min. By means of a switch valve system, a positive pressure was generated in the vial, which drove the volatiles onto the capillary column that had been preset to 40°C. The oven then was heated by 10°C/min to 135°C. Volatiles were detected by a flame ionization detector and peaks of the compounds were recorded.

**Figure 2:**
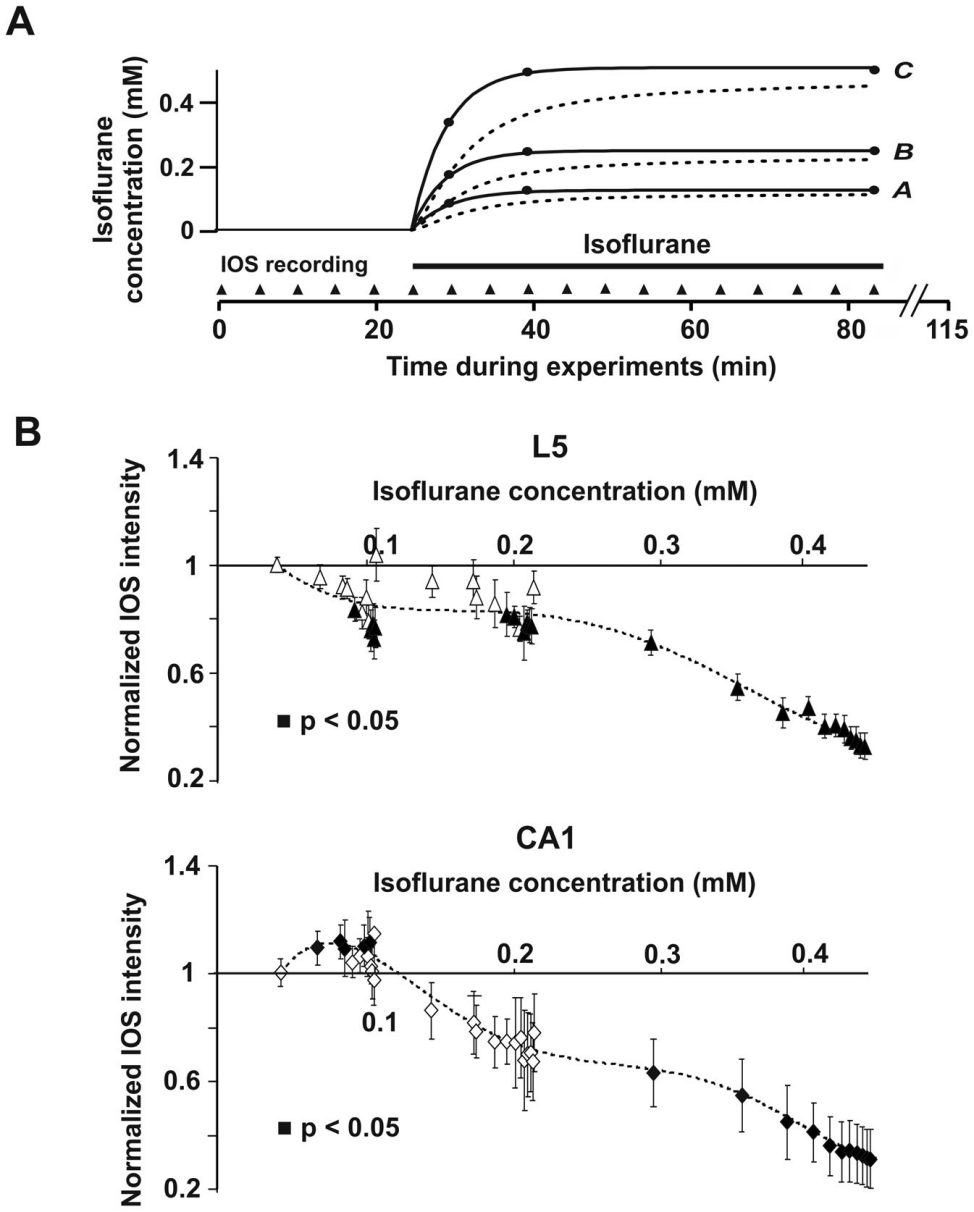
Relation between isoflurane concentration and NNE as measured by IOSs. (A) Isoflurane bath concentrations during the experiments measured by gas chromatography (closed circles, solid lines) and isoflurane tissue concentrations modeled via Fick’s diffusion law (dotted lines) for the three different vapor settings (*A*, *B*, and *C*) used. Isoflurane has to enter the brain slice submerged in the recording chamber via diffusion, causing a delayed equilibration of bath and tissue concentrations. (B) Dose-response curves calculated using the isoflurane tissue concentrations from (A) and the measured IOS intensities depicted in Figure 3. 0.1 mM isoflurane increases the IOS in the CA1 region of the hippocampus, while it decreases it in neocortical layer 5 (L5) at the same time. Isoflurane concentrations >0.3 mM considerably reduce the IOS in CA1 as well as in the neocortex.



Tertiary butanol in water served as internal standard (80 mg/l). Headspace vials (volume: 20 ml) were filled with sodium sulfate anhydrous (500 mg), water (500 µl), internal standard solution (100 µl) and aqueous samples (250 µl), and were immediately sealed with Teflon caps. The sample was equilibrated for 40 min at 60°C. A headspace sample was applied to the capillary column as mentioned above. From the ratios of the peaks of tertiary butanol (retention time 7 min 56 s) and isoflurane (retention time 7 min 17 s), isoflurane concentrations were derived by a calibration curve. To generate the calibration curve, known amounts of isoflurane (2 or 5 µl, respectively) were dissolved in methanol (5 ml) in a narrow-necked volumetric flask and ACSF was added to a total volume of 100 ml. Together with sodium sulfate anhydrous, water, and internal standard, these calibration solutions were analyzed as described above. For each of the three vapor settings *A*, *B*, and *C*, aqueous samples were withdrawn from the recording chamber at 5 min, 15 min, and 60 min after onset of delivery of isoflurane containing ACSF. The determined bath concentrations corresponding to the three vapor settings *A*, *B*, and *C* were 0.08 mM (*A*), 0.17 mM (*B*), and 0.33 mM (*C*) after 5 min, and 0.12 mM (*A*), 0.24 mM (*B*), and 0.49 mM (*C*) after 15 and 60 min, respectively. We derived the following exponential equations describing the isoflurane bath concentrations in mM at vapor settings *A*, *B*, and *C* from these values (**Figure 2A**).

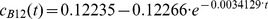
.
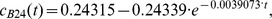


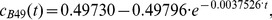
(1)


### Modelling of Isoflurane Tissue Diffusion

Diffusion processes are generally described by Fick’s 2^nd^ law:
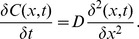
(2)(*D*: diffusion constant. *C*(*x*,*t*): concentration at location *x* and time *t*).

It was assumed that an exchange with the bath medium only takes place at the upper slice surface being exposed to the bath solution. Under this assumption solving eq. (2) with the boundary conditions *C*(0,*t*) = *c_B_*(*t*) and *C*(∞,*t*) = 0 yields to
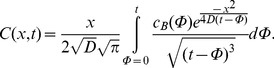
(3)(*t*: time after onset of isoflurane administration. *c*(*t*): isoflurane bath concentration. *x*: penetration depth within the slice).

We determined numerical solutions for (3) with respect to the three functions *c_B12_*(*t*), *c_B24_*(*t*), and *c_B49_*(*t*) describing the measured isoflurane bath concentrations at vapor settings *A*, *B*, and *C* (eq. 1). The diffusion constant of isoflurane was assumed as *D* = 1.6×10^−10^ m^2^/s. By integration over the whole slice thickness (300 µm) the average tissue concentrations were modelled as depicted in **Figure 2A**.

### Statistics

All values are expressed as the mean ± standard error of the mean (SEM). Only a single brain slice was used from each animal, and only a single experiment was carried out with each slice. All statistical evaluations were performed with the single-sample Student’s t-test.

### Software

Image processing, stimulus generation, application of drugs, and calculation of average signal intensities was done by custom-made software written in the Igor Pro programming language (WaveMetrics, Oregon, USA). Statistical calculations were done with SigmaPlot (Systat software, Germany). Modelling of isoflurane tissue diffusion was performed via Maple (Maplesoft, Canada).

## Results

**Figure 3:**
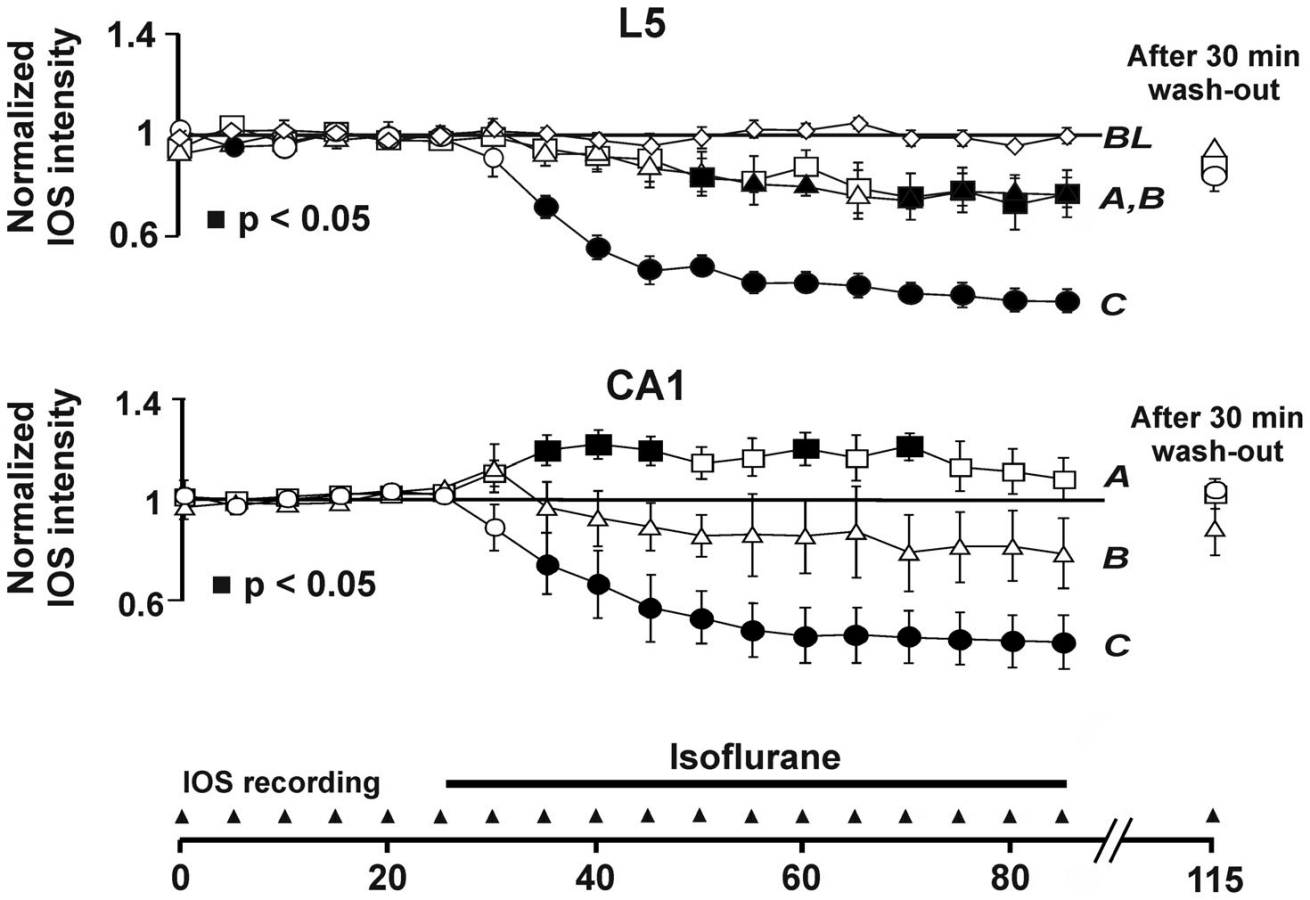
Summarized results from 22 experiments performed with three different isoflurane vapor settings. *A* (low dose, n = 7 brain slices), *B* (medium dose, n = 6 brain slices), and *C* (high dose, n = 9 brain slices). *BL*, baseline obtained without drug application (n = 3 brain slices). Start and end of isoflurane application are marked by the bar.

**Figure 4:**
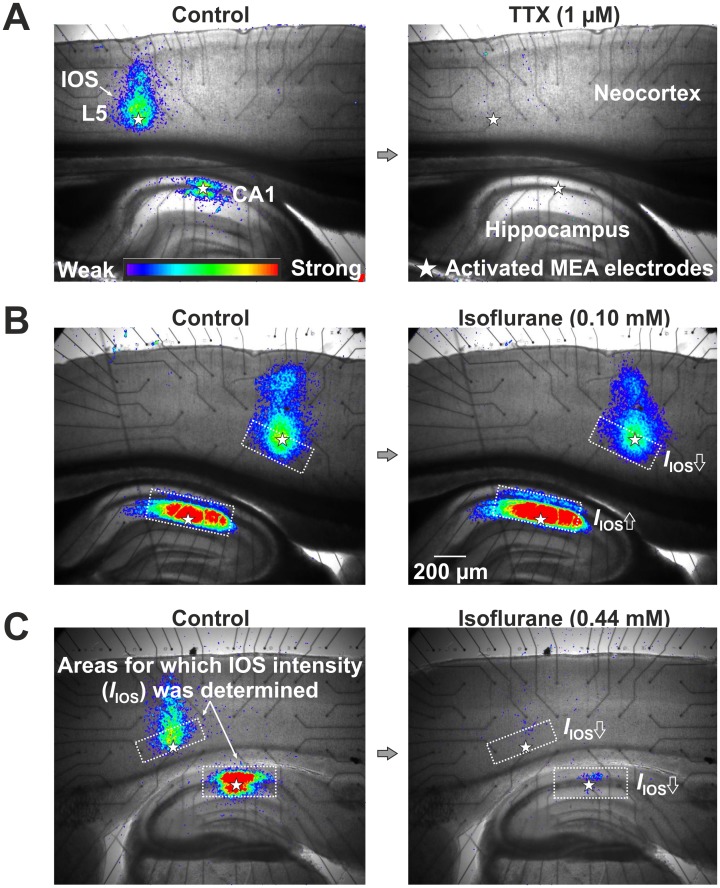
Recording of the IOS in hippocampal area CA1 and neocortical layer 5 (L5) in a rat brain slice. (A) Control experiment without isoflurane application (left). IOSs in the same brain slice after application of TTX (right). (B) IOSs in the absence and presence of 0.1 mM isoflurane. An amplification is clearly detectable in the hippocampus, but not in the neocortex. (C) Isoflurane (0.44 mM) markedly reduced the IOS in the hippocampus as well as the neocortex.

We investigated concentration-dependent actions of isoflurane in neocortical layer 5 and in the CA1 region of the hippocampus by quantifying the intensity of the IOS after electrical stimulation. To this end, we simultaneously applied 2 s long electrical pulse trains to the two brain regions via a MEA. Starting from the onset of stimulation, we recorded temporal sequences of ten IOS images with a time interval of 2 s. These measurements were repeated in 5 min intervals over a time span of totally 115 min. The intensity of the evoked IOSs was quantified by a computer program. To test the stability of control recordings, we performed six IOS measurements (min 0–25) before onset of drug application in each brain slice. As demonstrated in previous studies, the IOS remains constant for up to several hours, if a stable baseline once has been achieved and the stimulation frequency is moderate [11]. We confirmed this for our experimental conditions by control experiments without any drug application (**Figure 3**, *BL*). **Figure 4A** left depicts two typical IOSs in neocortex and hippocampus. As a further control we applied 1 µM of the sodium channel blocker tetrodotoxin (TTX). Expectedly, the IOSs were fully abolished, confirming that the measured signal depends on neuronal action potential firing (**Figure 4A** right).

Starting from min 25 of the experiments, we applied isoflurane for a time span of 60 min. Afterwards, the slices were superfused with isoflurane-free ACSF for further 35 min. We totally performed 22 experiments using three different vapor settings and, thus, three different isoflurane concentrations (vapor setting *A* (lowest): n = 7 brain slices; vapor setting *B*: n = 6 brain slices, vapor setting *C* (highest): n = 9 brain slices).

**Figure 4B and C** depict IOSs in the absence (control) and presence of 0.1 or 0.44 mM isoflurane. For quantitative analysis, we determined average IOS intensities within rectangular regions of interest (ROIs). The ROIs were set due to visual inspection so that they best-possibly covered the CA1 region of the hippocampus or neocortical layer 5, respectively, and least-possibly extended into neighbored structures. From **Figure 4B** an increase in the IOS intensity caused by low dose isoflurane (0.1 mM) is clearly detectable in the hippocampus, but not in the neocortex. **Figure 3** comprises the data from 22 experiments. In neocortical layer 5, a monotonous, concentration-dependent decrease in NNE as measured by the IOS intensity was observed for vapor settings *A*, *B*, and *C* (single-sample t-test with p<0.05).

We found a different isoflurane action on NNE in the hippocampus. Using the lowest vapor setting (*A*), a significant increase in the IOS intensity occurred after 10 min isoflurane application. Higher isoflurane concentrations applied using vapor settings *B* and *C*, however, decreased the IOS by a similar amount as in the neocortex (**Figure 3**). An initial transient increase in NNE, which was followed by a decrease, was observed in most experiments performed with the intermediate vapor setting *B*.

Using gas chromatography, we determined the isoflurane bath concentrations corresponding to the different vapor settings (see Methods). From the results, equations describing the rise of the isoflurane bath concentrations with time after vapor activation were derived (**Figure 2A**). Isoflurane solved in the bath solution enters a submerged brain slice passively via diffusion mainly at the upper surface, hence causing a delayed equilibration of isoflurane concentrations in the neuronal tissue. Since it is not practical to measure isoflurane concentrations directly in the brain slice, we modeled the expected time courses of isoflurane concentrations in the brain tissue utilizing Fick’s diffusion law (**Figure 2A**) (see Methods).

Using the determined isoflurane tissue concentrations, we were able to derive dose-response curves for the measured isoflurane effects on NNE. **Figure 2B** shows that a concentration of 0.1 mM isoflurane significantly enhances NNE in the CA1 region of the hippocampus, while it simultaneously decreases it in cortical layer 5 (p<0.05, single-sample t-test). At concentrations >0.3 mM, a significant decrease in NNE was found for both brain regions investigated.

## Discussion

General anesthetics are able to produce clinical excitation. This still enigmatic and potentially harmful anesthetic state, which can precede and/or follow deep surgical anesthesia is marked by a coincidence of behavioral excitation and unconsciousness [1], [14]. These symptoms give reason to speculate that general anesthetics, at slightly narcotic concentrations, may increase activity in some part(s) of the CNS, while simultaneously decreasing it in other CNS structures. We provide evidence for this hypothesis by demonstrating *in vitro* that the volatile general anesthetic isoflurane can concurrently increase and decrease NNE in the hippocampal CA1 region and neocortical layer 5, respectively. Such a mechanism may help to understand the symptoms of behavioral arousal, although consciousness and the ability for targeted actions are blocked.

Previous studies clearly show that IOSs represent a valid measure of NNE. In particular, the generation of IOSs depends on excitatory synaptic transmission as well as spiking of neurons [10], [15]. Consistently, the GABA_A_ receptor agonist muscimol decreases IOSs, while the GABA_A_ antagonist bicuculline markedly enhances them [15]. In addition, a high correlation between spatial distributions of field potentials and the spatial distribution of IOSs was proven by different groups [11], [16], [17].

We consciously chose the IOS technique, as we attempted to study isoflurane effects at the neuronal network level. Neither field potential recordings, nor patch-clamp experiments are suited to perform that way, since they only provide a local measure of the spiking activity of a very limited population of neurons. It has repeatedly been shown previously that isoflurane markedly decreases field excitatory postsynaptic potential and population spike amplitudes in area CA1 in rodent brain slices [18], [19]. The strength of these effects, as well as their kinetics, is similar to those we observed in our IOS recordings. MacIver and Roth [18] also reported an enhancement of neuronal activity in the CA1 region with low-dose isoflurane using classical electrophysiological methods.

It has been confirmed in multiple studies that volatile anesthetics increase GABAergic inhibition [20], [21] and decrease glutamatergic synaptic transmission [22], [23]. Both mechanisms presumably contribute in parallel to the reduction in brain activity associated with deep anesthesia [8], [14], [23]–[25]. This corresponds well to the results of our experiments performed with high isoflurane doses. However, the enhancement of the IOS by low dose isoflurane cannot be explained by these mechanisms and needs further investigation to become unraveled.

We have chosen isoflurane for our experiments as test substance because it is a commonplace general anesthetic known to induce an excitement phase also in rats [7]. As a reliable measure of NNE in brain slices, we used the intensity of the IOSs that were evoked by electrical stimulation of neocortical layer 5 and hippocampal area CA1 [9]–[11], [26]. These brain regions represent important output structures of the neocortex and hippocampus.

To our knowledge, only one study exists in which sub-narcotic doses of a volatile anesthetic have been found to increase neuronal excitability *in vitro* [18]. In this work, a transient augmentation of population spike amplitudes, followed by suppression with increasing doses of isoflurane, was found in the CA1 region of the hippocampus. The dose applied for augmentation was 0.5 VOL% (0.13 mM), which is almost identical to the lowest bath concentration applied in our experiments.
